# Neem Leaf Glycoprotein Activates CD8^+^ T Cells to Promote Therapeutic Anti-Tumor Immunity Inhibiting the Growth of Mouse Sarcoma

**DOI:** 10.1371/journal.pone.0047434

**Published:** 2013-01-11

**Authors:** Atanu Mallick, Subhasis Barik, Kuntal Kanti Goswami, Saptak Banerjee, Sarbari Ghosh, Koustav Sarkar, Anamika Bose, Rathindranath Baral

**Affiliations:** Department of Immunoregulation and Immunodiagnostics, Chittaranjan National Cancer Institute (CNCI), Kolkata, India; National Institute of Allergy and Infectious Diseases, United States of America

## Abstract

In spite of sufficient data on Neem Leaf Glycoprotein (NLGP) as a prophylactic vaccine, little knowledge currently exists to support the use of NLGP as a therapeutic vaccine. Treatment of mice bearing established sarcomas with NLGP (25 µg/mice/week subcutaneously for 4 weeks) resulted in tumor regression or dormancy (Tumor free/Regressor, 13/24 (NLGP), 4/24 (PBS)). Evaluation of CD8^+^ T cell status in blood, spleen, TDLN, VDLN and tumor revealed increase in cellular number. Elevated expression of CD69, CD44 and Ki67 on CD8^+^ T cells revealed their state of activation and proliferation by NLGP. Depletion of CD8^+^ T cells in mice at the time of NLGP treatment resulted in partial termination of tumor regression. An expansion of CXCR3^+^ and CCR5^+^ T cells was observed in the TDLN and tumor, along with their corresponding ligands. NLGP treatment enhances type 1 polarized T-bet expressing T cells with downregulation of GATA3. Treg cell population was almost unchanged. However, T∶Treg ratios significantly increased with NLGP. Enhanced secretion/expression of IFNγ was noted after NLGP therapy. *In vitro* culture of T cells with IL-2 and sarcoma antigen resulted in significant enhancement in cytotoxic efficacy. Consistently higher expression of CD107a was also observed in CD8^+^ T cells from tumors. Reinoculation of sarcoma cells in tumor regressed NLGP-treated mice maintained tumor free status in majority. This is correlated with the increment of CD44^hi^CD62L^hi^ central memory T cells. Collectively, these findings support a paradigm in which NLGP dynamically orchestrates the activation, expansion, and recruitment of CD8^+^ T cells into established tumors to operate significant tumor cell lysis.

## Introduction

Immune mediated restriction of tumor growth essentially requires synchronization of several interdependent events, including activation of tolerized immune cells [1], their migration and homing [2], suppression of suppressor activities of regulatory cells [3], type 1 polarization of immune microenvironment [4], inhibition of interference of pro-tumor molecules [5], memory development to prevent recurrence [6] and normalization of tumor vasculature [7]. Among these events, effector CD8^+^ T cells might occupy the key position in cancer immunotherapeutic approaches [8] though these cells are frequently anergic or apoptotic in such situation [9]. Adoptive T cell therapy after their *ex vivo* expansion is increasingly developing into a subject of interest in cancer clinical trials [8]. The most remarkable results thus far have been produced by T cell transfer for metastatic melanoma and the combination of surgery and adoptive T cell therapy for hepatocellular carcinoma [10], [11]. However, the ability of transferred CD8^+^ cytotoxic T cells (CTLs) to recognize tumor antigens is an essential requirement that may not be always possible in *ex vivo* expansion.

As carcinogenesis initiated and progressed, several regulatory mechanisms (mediated by regulatory T cells (Tregs), tumor associated macrophages (TAMs), myeloid derived suppressor cells (MDSCs)) turn out to be dynamic and maintain immune tolerance within tumor microenvironment (TME) to negatively interfere with CD8^+^ T cell functions [12], [13]. Poor tumor homing and penetration of effector T cells, a consequence of aberrant vasculature and limited chemokine expression, is another major barrier to antitumor immunity [14]. Systemic immunity is affected to a variable degree, but immune suppression is typically most profound within the TME. Accordingly, CD8^+^ T-cells exhibited poor cytotoxic function [15]. In designing effective immunotherapy [16] and to obtain better clinical outcome [14], substantial emphasis has recently been placed on the development of treatment modalities that are capable of restoring systemic and tumor infiltrated T-cell functions [17] and associated immune dysfunctions [18].

In prophylactic settings, we have reported that Neem Leaf Glycoprotein (NLGP), a nontoxic preparation from neem (*Azadirachta indica*) leaf, can effectively prevent the murine tumor growth [19], [20]. NLGP can activate T cells [21], [22], NK cells [23], inhibit Tregs [24], promote type 1 cytokine microenvironment [25], [26] and rectify altered chemokine signaling [27], [28], thereby, inducing antigen-specific tumor cell killing [19]–[21], [29]. Here, for the first time, we have tested the therapeutic potential of NLGP in sarcoma tumor model. Understanding the mechanism of observed tumor growth restriction is attempted in light of CD8^+^ T cell activation and associated tumor suppressive functions.

## Results

### NLGP Therapy provides Superior Anti-Tumor Efficacy against Sarcoma

**Figure 1:**
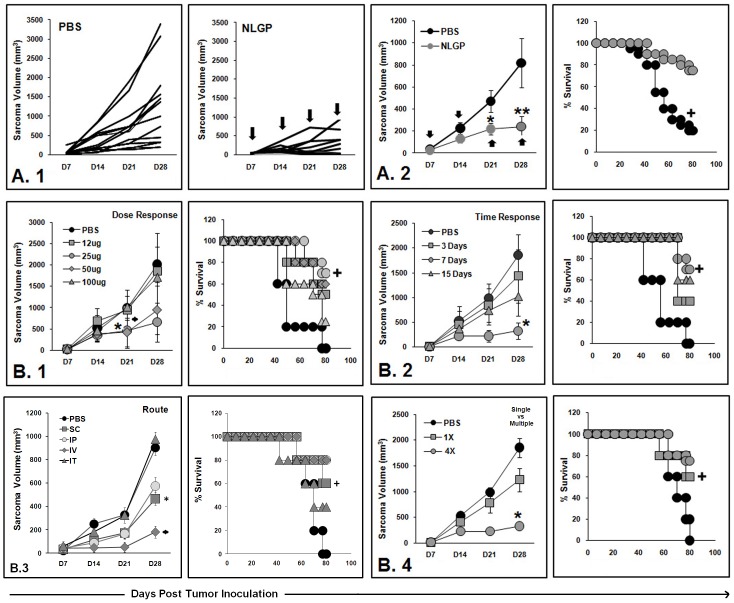
Tumor growth and survivability of NLGP-treated mice. **A.1**. Swiss mice were inoculated with Sarcoma 180 cells (1×10^6^ cells/mice). After formation of palpable tumor, mice of experimental group (*n* = 24) were treated with NLGP (25 µg) once a week for 4 weeks in total and other control group (*n* = 24) received PBS only. Pattern of tumor growth (in mm^3^) of each mouse is presented till day 28 after the initiation of therapy. Arrows indicate the points of NLGP injection **A.2**. Mice were inoculated with s.c. sarcoma and after formation of tumors mice of experimental group were treated with NLGP (25 µg) once a week for 4 weeks in total. Mean tumor volume of PBS- and NLGP-treated mice is presented (*n* = 24, in each case) (*p<0.05; **p<0.001), along with their mean survival till day 90 (^+^ *p*<0.001). **B.1**. Mice were inoculated with Sarcoma 180, after formation of palpable tumors, mice were randomly divided into five groups (*n* = 6, in each group) and treated with different doses of NLGP (12 µg, 25 µg, 50 µg and 100 µg) once a week for 4 weeks in total and PBS for fifth group. Tumor growth curve (*p<0.01 for 25 µg; ^♦^ *p*<0.01 for 50 µg) and their mean survival are presented (^+^ *p*<0.01). **B.2**. Mice were inoculated with Sarcoma 180; after the formation of the palpable tumors, mice were randomly grouped into four (*n* = 6, in each group) and treated with NLGP (25 µg) s.c. for various time intervals (3 days, 7 days and 15 days) and 4 injections were given in each treatment cohort. Fourth group was kept untreated. Tumor growth curve (*p<0.001) and their mean survival (^+^ *p*<0.01) are presented. **B.3**. Mice were inoculated with Sarcoma 180; after the formation of the palpable tumors, mice were randomly grouped into five (*n* = 6, in each group) and treated with NLGP, through different routes (subcutaneous, intraperitoneal, intravenous and intratumoral) once a week for 4 weeks in total. Tumor growth curve (*p<0.01, s.c. and i.p.; ^♦^ *p*<0.01, iv) and their mean survival (^+^ *p*<0.01) are presented. **B.4**. Mice were inoculated with Sarcoma 180; after the formation of the palpable tumors, mice were randomly grouped into three (*n* = 6, in each group) and treated with NLGP (25 µg) (single vs four weekly injections). Tumor growth curve (*p<0.001) and their mean survival (^+^ *p*<0.01) are presented.

Mice harboring established day 7 sarcoma were left untreated or they were administered with NLGP (25 µg) weekly for 4 weeks in total. Result of such treatment schedule on sarcoma bearing mice is shown in Figure 1A.1 and Table 1, which yielded significant protection against sarcoma progression. This result shows progression of the disease in 11/24 (45.8%) NLGP-treated mice as compared to 20/24 (83.3%) mice in untreated control groups. Moreover, tumor volume in NLGP-treated mice (s.c., weekly for 4 weeks) with progressive disease is significantly less than PBS controls (Figure 1A.2). Again, the number of regresser plus tumor free animals is significantly more in mice group that received NLGP once a week for 4 weeks in total through s.c. route (13/24 (54.2%)) than those that received PBS only (4/24 (16.6%)). Notably, NLGP-treated cohort contained regressor animals, with 41.6% (i.e. 10/24) of mice rendered tumor-free by 60 days post-tumor inoculation. In PBS group only 16.6% (i.e. 4/24) mice remained tumor free till day 60. Survival benefit of NLGP-treated mice is 90% (22/24) on day 60, vs 25% (6/24) in PBS group (*p*<0.001) (Figure 1A.2).

**Table 1:** Disease status of sarcoma bearing mice with NLGP^1^ therapy.

Treatments								
	NLGP				PBS			
Disease Status	No.	%	MTV*	MS+	Number	%	MTV	MS
**Progressive**	11	45.8	677.9	50	20	83.3	983.6	25
**Regressive**	3	12.5	32.0	100	0	0.0	-	-
**Tumor Free**	10	41.7	-	100	4	16.7	-	100
**Total**	24	100	-	-	24	100	-	-

MTV, Mean Tumor Volume on day 28;

* in mm^3^.

MS, Mean survival on day 70;

+ % of mice.

1 25 µg/mice/week for 4 weeks.

The impact of NLGP dose alteration on the anti-tumor efficacy was determined next. As shown in Figure 1B.1, NLGP doses of 25 and 50 µg/mice resulted in a significant improvement of the anti-tumor effectiveness (*p*<0.01 for 25 µg and 50 µg doses on day 21). However, minimum restriction was noted in the other dose groups. The time interval of NLGP administration also appeared important, with superior efficacy demonstrated in injection interval of 7 days than 3 and 15 days interval (Figure 1B.2). Comparative analysis on NLGP immunization by different routes revealed best tumor restriction by intravenous injection; however, considerable ability of other routes proved worthy (Figure 1B.3). For easy delivery, subcutaneous route was used here. Again, data provided in Figure 1B.4 suggest a major anti-tumor benefit of NLGP in repeated vaccination over single vaccination (*p*<0.01).

### NLGP Therapy provides Superior CD8^+^ T Cell Responses

**Figure 2:**
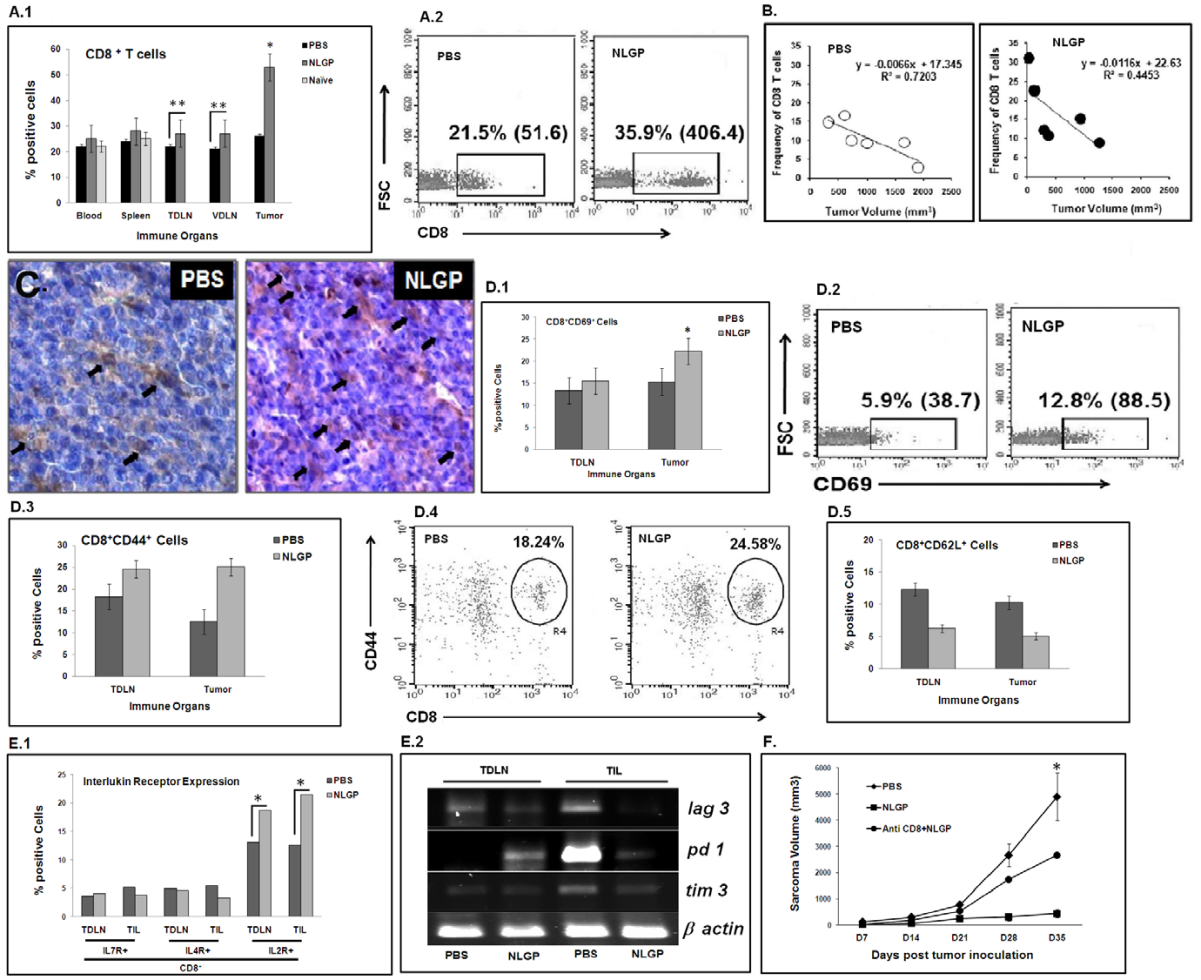
NLGP mediated tumor growth restriction and CD8^+^ T cells. Mice were inoculated with Sarcoma 180 tumor cells (1×10^6^ cells/mice); after the formation of palpable tumor, mice were treated with NLGP (25 µg) once in a week, and four injections in total. **A.1**. Different immune organs (Blood, Spleen, TDLN, VDLN) and tumors were harvested on day 21 after initiation of therapy, lymphocytes were isolated, labeled with CD8-PE and percentage of CD8^+^ T cells were analyzed by flow cytometry, along with blood and spleen cells from naive mice. *p<0.001; **p<0.05. **A.2**. Representative dot plots of CD8 positivity of TILs harvested from tumor of PBS- and NLGP-treated mice. **B**. Tumor volume of PBS- and NLGP-treated mice was plotted against percentage of CD8^+^ cells. **C**. Presence of CD8^+^ T cells was detected on cryosections of tumor tissues from either PBS or day 21 NLGP-treated mice by immunohistochemistry. **D.1**. MNCs from TDLN and tumors were harvested from mice with day 21 tumors and stained for CD8^+^CD69^+^. *p<0.05. **D.2**. CD8^+^ cells within MNCs of PBS- and NLGP-treated mice were gated and CD69^+^ cells were identified within this gated population as presented by dot plots. **D.3**. MNCs from TDLN and tumors were harvested from mice with day 21 tumors and stained for CD8^+^CD44^+^. **D.4**. A representative dot plot for CD8^+^CD44^+^ cells within MNCs of PBS- and NLGP-treated mice. **D.5**. MNCs from TDLN and tumors were harvested from mice with day 21 tumors and stained for CD8^+^CD62L^+^ cells. **E.1**. Expression of cytokine receptors, like, IL-7R, IL-4R and IL-2R, on cells from TDLN and TIL of NLGP-treated mice was determined flow cytometrically. **E.2**. Expression of T cell exhaustion markers, like, lag3, pd1 and tim3, in cells from TDLN and TIL of NLGP-treated mice was determined at transcriptional level by RT-PCR. **F**. CD8^+^ cells were depleted in NLGP-treated mice, along with PBS (negative) and NLGP (positive) treatment into two other groups as controls. Tumor growth curve is presented. *p<0.001, in comparison to NLGP-treated group.

Above results pointed out that NLGP treatment following s.c. sarcoma inoculation (1×10^6^ cells/mice) caused significant tumor growth restriction. In order to elucidate the involvement of CD8^+^ T cells in such restriction, we checked the CD8^+^ T cell status within blood, of spleen, tumor draining lymph nodes (TDLN), vaccine draining lymph nodes (VDLN) and tumor by harvesting cells on day 21 post-tumor inoculation. Flow cytometric evidences suggest that the number of CD8^+^ T cells was more within the cells from tumor of NLGP-treated mice than PBS control. (Figure 2A.1 and A.2). Interestingly, the number of CD8^+^ T cells within TME was inversely proportional to the extent of tumor growth (Figure 2B). Immunohistochemical analysis also supports the increment of CD8^+^ T cells within tumor (Figure 2C). Activation status of existing CD8^+^ T cells in TDLNs and tumors was studied by co-staining them with anti-CD69, anti-CD44 and anti-CD62L antibodies, where upregulation of CD69 and CD44 positive cells with downregulation of CD62L was documented within CD8^+^ cells from lymphocytes in TDLN and tumors (Figure 2D.1–D.5).

Phenotypic expression of interleukin receptors (IL-7R, IL-4R and IL-2R) was studied on T cells from tumors of PBS- and NLGP-treated mice. No significant change was noticed on expression of IL-7R, IL-4R. However, significant upregulation in IL-2R expression was observed (Figure 2E.1). Within TME, activation of T cells is generally followed by exhaustion of these cells, which imparts negative effect on anti-tumor T cell immunity. We have studied three genes associated to T cell exhaustion and all of them (lag3, pd1 and tim3) were downregulated in tumor infiltrating lymphocytes from NLGP-treated mice (Figure 2E.2). To further confirm the role of CD8^+^ T cells in tumor growth restriction following NLGP therapy, mice were depleted for these particular cells using specific neutralizing antibody. CD8 depletion following tumor inoculation resulted in increase in tumor growth in NLGP-treated mice (*p*<0.01), suggesting that NLGP mediated tumor growth inhibition is CD8^+^ T cell dependent (Figure 2F). Survival benefit of NLGP therapy was not found following *in vivo* CD8 depletion in mice *(data not shown)*.

### NLGP Therapy Elicits Superior Antigen-Specific Type-1 CD8^+^ T Cell Responses

**Figure 3:**
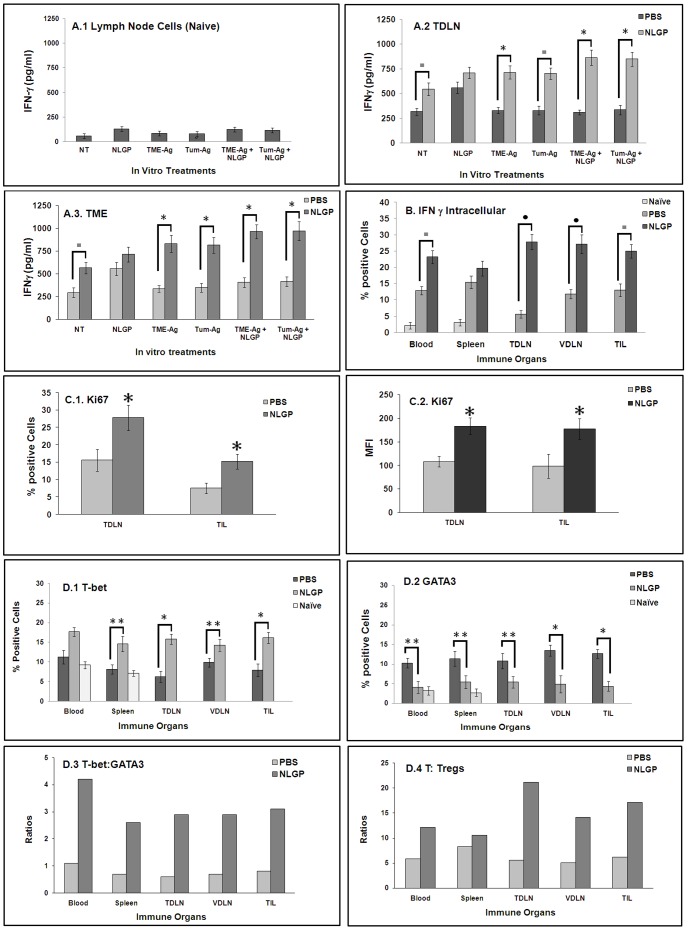
IFNγ secretion from immune cells of NLGP-treated tumor bearing mice. Mice were inoculated with Sarcoma 180 cells (1×10^6^ cells/mice) and after the formation of palpable tumor mice of experimental group were treated (s.c.) with NLGP once a week. Mice were sacrificed on day 21 to collect TDLN and tumor and MNCs were purified. **A**. Cells from lymph nodes of naïve mice (A.1), TDLNs (A.2) and tumors (A.3) were cultured *in vitro* for 48 h in presence of NLGP, TME-Ag, Tum-Ag, TME-Ag+NLGP, Tum-Ag+NLGP in RPMI 1640. Culture supernatants were assessed for IFNγ release by ELISA. *p<0.01; ^▪^ *p*<0.05. **B**. Intra-cellular IFNγ expression of CD8^+^ T-cells in different immune compartments (from either PBS- or NLGP-treated group and from blood and spleen cells of naïve mice) was analyzed by flow cytometry. ^•^ *p*<0.01; ^▪^ *p*<0.05. **C**. MNCs isolated from TDLN and TIL of PBS- and NLGP-treated mice were labeled to detect the proliferation specific marker, Ki67, along with CD8^+^ cells. Percentage of proliferated cells within CD8 gated population and their MFI were presented. *p<0.01. **D**. Mice were inoculated with Sarcoma 180 cells (1×10^6^ cells/mice) and after formation of palpable tumor, mice of experimental group were treated with NLGP once a week. Mice were sacrificed on day 21 to collect blood, spleen, TDLN, VDLN and tumor and CD8^+^ T cells were purified from MNCs. Blood and spleen cells were also harvested from naive mice. Purified CD8^+^ T cells were intracellularly stained with anti-T-bet (**D.1**) and anti-GATA3 (**D.2**) antibodies to analyze through flow cytometry. Percent of T-bet and GATA3 positive cells from different immune organs is presented. *p<0.001; **p<0.01. Ratio between T-bet vs GATA3 (**D.3**) and T cells vs Tregs (**D.4**) is shown.

Confirmation on the CD8^+^ T cell dependence in NLGP mediated therapeutic tumor growth restriction prompted us to unravel such mechanism. Next, we studied antigen specific T cell response, by assessing their ability to secrete/express IFNγ from T cells present in MNCs from blood, spleen, TDLN, VDLN and tumor of day 21 sarcoma bearing mice. These mice with therapeutic intervention of NLGP always showed greater extracellular (Figure 3A) and intracellular (Figure 3B) IFNγ release. In response to further *in vitro* stimulation with tumor antigen (Tum-Ag) and tumor microenvironmental antigen (TME-Ag), there is enhanced IFNγ secretion with or without NLGP supplementation (Figure 3A). Negligible IFNγ release was observed from lymph node cells of naïve mice following antigenic stimulation (Figure 3A.1). Proliferating ability of T cells was checked by labeling these cells with proliferation marker Ki67. Significantly higher trend of proliferation was noted in day 21 sarcoma bearing mice under NLGP therapy (*p*<0.01) (Figure 3C).

To further confirm the type 1 convergence, we have analyzed the expression of Type 1 specific transcription factor, T-bet. Intracellular assessment revealed steady increase in number of T-bet expressing cells in mononuclear cells (MNCs) from blood, spleen, TDLN, VDLN and tumor of day 21 sarcoma bearing mice (Figure 3D.1). Expression per cell is also upregulated particularly in TDLN and TILs. On the other hand, cells with GATA3 expression appeared to be few in different immune compartments from sarcoma bearing NLGP-treated mice (Figure 3D.2). As shown in Figure 3D.3, Tbet∶GATA3 ratios are significantly greater in NLGP-treated sarcoma mice.

In association with type 1 T-cell polarization, effective cancer immunotherapies have been largely associated with an increased T effector-to-Treg ratio [30], [31]. In examining the status of regulatory cells, we have observed no significant changes in CD4^+^CD25^+^Foxp3^+^ T regulatory cells in various immune compartments in day 21 NLGP-treated sarcoma bearing mice (*data not shown*). As optimum therapeutic outcome may depend on the balance between effector-to-regulatory T cell ratio, next, we determined the ratio between CD8^+^ effector T cells and CD4^+^CD25^+^ regulatory T cells. Results as presented in Figure 3D.4 revealed increment in such ratio, favoring the T cell mediated anti-tumor immunity. Other regulatory molecules, such as IL-10, TGFβ, IDO, were also downregulated in tumors of NLGP-treated sarcoma mice *(data not shown)*.

### NLGP Promoted Expression of CXCR3/CCR5 and Corresponding Ligands may Direct T Cells to TME

**Figure 4:**
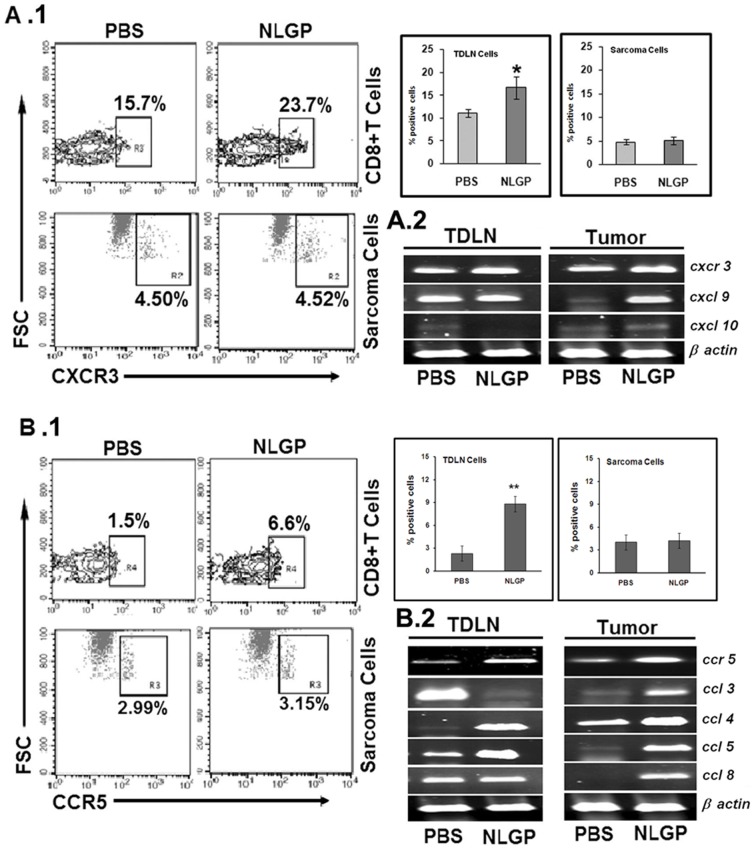
CXCR3, CCR5 expression on CD8^+^ T cells (from TDLN) and sarcoma cells. Mice were inoculated with Sarcoma 180 cells (1×10^6^ cells/mice) and after formation of palpable tumor mice of experimental group were treated with NLGP (25 µg) once a week s.c. for 4 weeks in total. Mice were sacrificed on day 21 to collect TDLN and MNCs were purified. CD8^+^ T cells *(upper panel, low FSC)* and sarcoma cells *(lower panel, high FSC)* with or without NLGP treatment were stained with anti-CXCR3 (**A.1**) and anti-CCR5 (**B.1**) antibodies. Representative dot plots in each case *(left panel)* and bar diagrams *(right panel)* showing mean±SD values of six individual observations are presented. *p<0.01; **p<0.05. Total RNA was isolated from T cells of TDLN and tumor of the same PBS- and NLGP-treated mice and cxcr3 and its ligands, e.g., cxcl9, cxcl10 were analyzed at transcriptional level (**A.2**). Similarly, mRNA for ccr5 and corresponding ligands, e.g., ccl3, ccl4, ccl5 and ccl8, were also assessed (**B.2**).

Earlier discussion demonstrated the recruitment of enhanced number of type 1 polarized CD8^+^ T cells within TME. Such recruitment to tumor sites occurs via CXCR3 mediated chemotaxis in response to the CXCL9-11 chemokines, produced within the TME [32]. Accordingly in order to determine whether TDLN/Tumor derived T cells in NLGP-treated animals are expressing more chemotactic components over PBS mice, we assessed CD8^+^ T cells for their expression of CXCR3 and corresponding ligands on day 21 sarcoma mice. As shown in Figure 4A.1, we found that CXCR3^+^ subpopulation of CD8^+^ T cells as well as cxcr3 transcripts were significantly increased in TDLN of NLGP-treated mice. Expression of mRNAs corresponding to cxcl9 and cxcl10 also increased within TILs from tumors (Figure 4A.2).

We also found upregulation of CCR5^+^ cells and enhanced ccr5 expression at mRNA levels (Figure 4B.1 and B.2). Though CCR5 is expressed generally on monocytes, it is also involved in T cell migration [33]. Consistently, we found higher expression of its corresponding ligands, ccl3, ccl4, ccl5 and ccl8 within TME. Similar result was obtained in case of TDLN for ccl4 and ccl5, without noticeable upregulation in ccl3 and ccl8 (Figure 4B.2). Interestingly, CXCR3 and CCR5 expression was unchanged on sarcoma cells upon *in vitro* NLGP treatment (Figure 4A.1 and B.1). These data suggest that NLGP therapy not only stimulates T cell expansion, but also licenses these cells for trafficking to peripheral tissues in which CXCR3/CCR5 ligands are expressed, such as TME.

### NLGP Therapy Elicits Superior Antigen-Specific T Cell Cytotoxic Response

**Figure 5:**
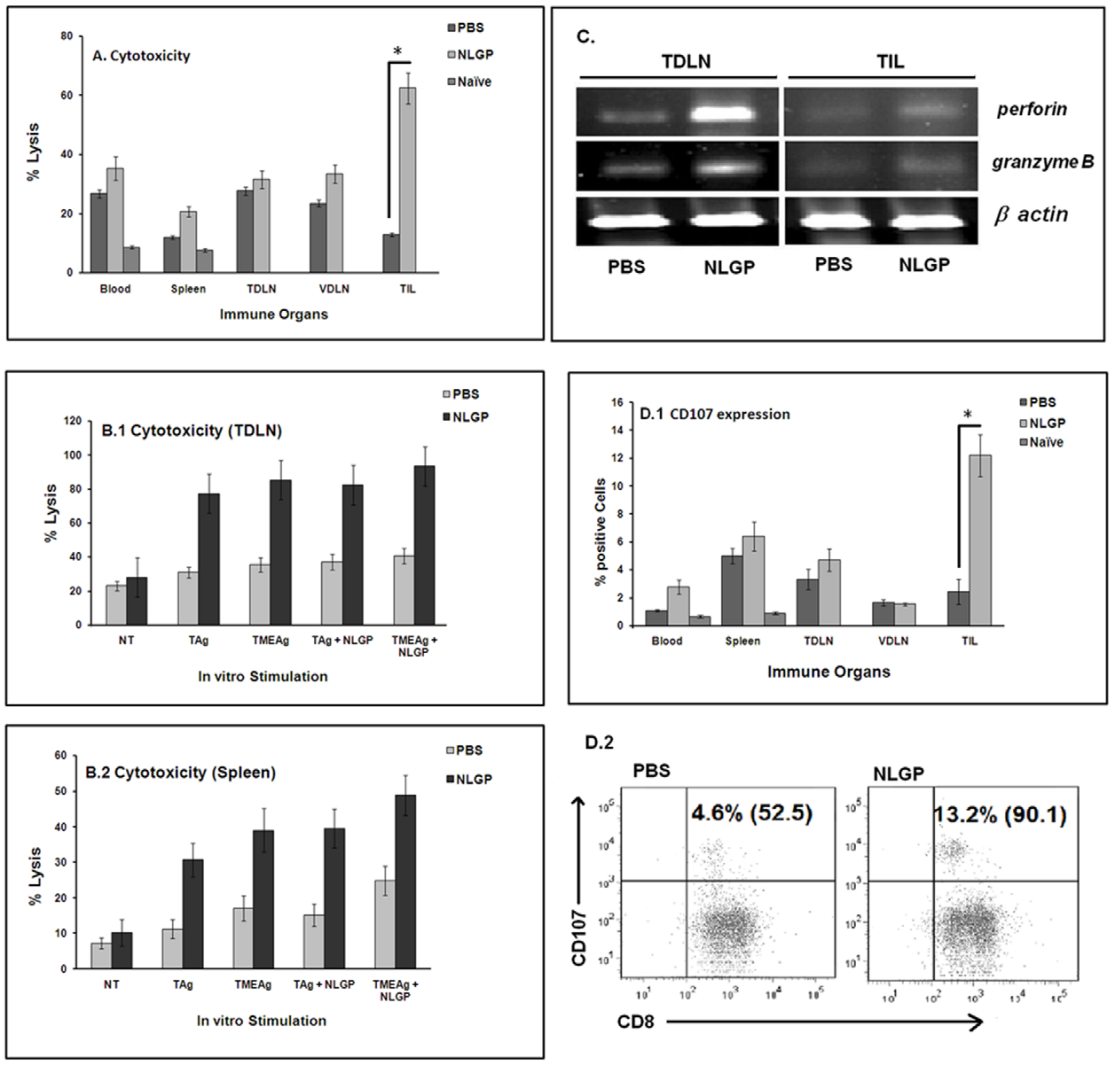
*In vitro* cytotoxicity of sarcoma by immune cells from NLGP-treated mice. Mice were inoculated with Sarcoma 180 cells (1×10^6^ cells/mice) and after formation of palpable tumor, mice of experimental group were treated with NLGP once a week. Mice were sacrificed on day 21 to collect blood, spleen, TDLN, VDLN and tumor and MNCs were purified. MNCs are also purified from blood and spleen of naive mice. MNCs were co-cultured with sarcoma cells in 1∶10 ratios for 24 hrs and released LDH was measured (**A**). *p<0.001. MNCs from TDLN (**B.1**) and Spleen (**B.2**) of PBS- and NLGP- treated sarcoma bearing mice were cultured *in vitro* in presence of IL-2 for 4 days and further stimulated with tumor (sarcoma) antigens (5 µg/ml) for 2 days with or without NLGP. Antigens from tumor as well as its microenvironment were extracted from intra-peritoneally grown sarcoma (Tum-Ag) and from solid tumor (TME-Ag). Cytotoxic efficacy of activated T-cells was analyzed towards Sarcoma 180 cells by LDH release assay. **C**. Total RNA was isolated from MNCs of TDLN and tumor from PBS- and NLGP-treated mice on day 21 and cytotoxicity related genes (perforin and granzymeB) were analyzed at transcriptional level by RT-PCR. **D**. MNCs from all immune compartments were incubated with sarcoma cells to detect CD107a expression by intracellular flow cytometric staining. Data is presented in bar diagram (*p<0.001) (**D.1**) and a representative dot plot for CD107a expression from TILs is also shown (**D.2**).

To explain the underlying facts of *in vivo* NLGP mediated tumor growth restriction, MNCs containing T cells were harvested from different immune compartments and incubated *in vitro* with sarcoma cells to observe their cytotoxic efficacy. T cells from blood, spleen, VDLN and TIL (TME) (*p*<0.001) (Figure 5A) of NLGP-treated mice have shown significantly greater cytotoxicity to sarcoma cells. However, cells from TDLN of NLGP-treated mice have comparable cytotoxicity with untreated control (Figure 5A).

To further clarify this point, MNCs from TDLN and spleen of NLGP-treated sarcoma bearing mice was cultured *in vitro* in presence of IL-2 for 4 days and further stimulated with Tum-Ag/TME-Ag (5 µg/ml in each case) for 2 days. Antigens from both sources elicited antigen specific T cell cytotoxic response (Figure 5B.1). TME-Ag generated greater cytotoxicity over Tum-Ag. Similar trend was detected when spleen cells were used (Figure 5B.2). Minimum cytotoxic reaction was noticed when antigen negative lymphoma was used *(data not shown)*.

In this context, RT-PCR analysis for cytotoxic genes was performed and noticeable upregulation of perforin and granzymeB was observed (Figure 5C). Moreover, as a result of interaction between CD8^+^ T cells and sarcoma cells, higher number of CD107a (an important cytotoxicity related molecule) was expressed on CD8^+^ T cells from different immune organs of NLGP-treated sarcoma mice, in comparison to untreated control. However, CD107a expression was remarkably higher on TILs within TME from NLGP mice (*p*<0.001), postulating the basis of tumor restriction (Figure 5D.1 and D.2).

### NLGP Therapy Elicits Anti-Tumor Central Memory Response to Prevent Tumor Growth following Sarcoma Re-Inoculation

**Figure 6:**
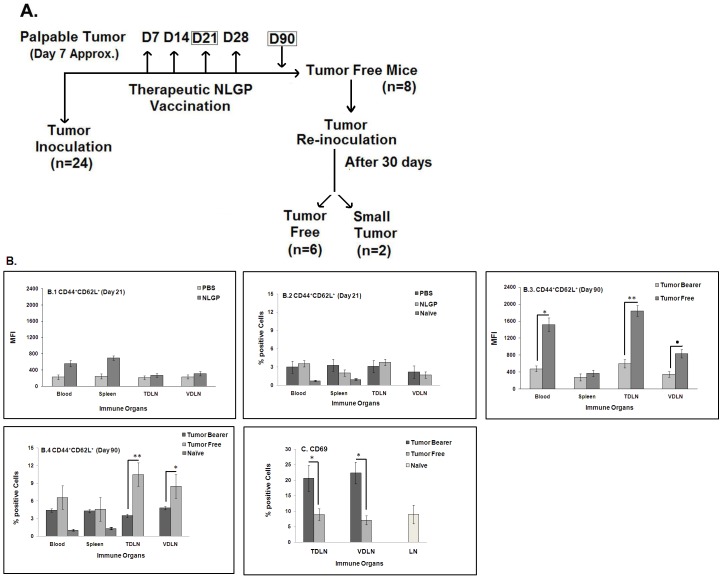
Memory response in sarcoma bearing mice after NLGP therapy. **A**. A flow chart explaining the experimental design to understand the generation of memory response following NLGP therapy and re-inoculation with sarcoma in tumor free mice. Mice were inoculated with Sarcoma 180 cells and after formation of palpable tumor mice of the experimental group were treated with NLGP (25 µg) once a week for 4 weeks in total. A group of mice was sacrificed on day 21 to collect blood, spleen, TDLN and VDLN. MNCs were purified to stain with CD44 and CD62L antibodies, along with staining for CD8^+^ T cells, in comparison to cellular components obtained from PBS injected sarcoma bearing mice. Expression on blood, spleen and lymph node cells from naive mice was also studied. Both status of % positive cells (**B.2**) and MFI (**B.1**) are shown. Mice were inoculated with Sarcoma 180 cells and after the formation of palpable tumor, mice of experimental group were treated with NLGP (25 µg) once a week for 4 weeks in total. Tumor free mice were selected following 90 days of the initiation of therapy and re-inoculated with sarcoma. Blood, Spleen, TDLN and VDLN were isolated from representative mice of same group on day 90 and MNCs were purified to stain with CD44 and CD62L antibodies, in comparison to cellular components obtained from untreated sarcoma bearing mice. Both status of % positive cells (**B.4**) and MFI (**B.3**) are shown. In experiments described in B.1–B.4, CD8^+^ cells were first gated and CD44^+^CD62L^+^ cells within this population were then analyzed. *p<0.01; **p<0.001; ^•^ *p*<0.05. Percentage of CD69^+^ cells were analyzed within CD8^+^ cells from TDLN and VDLN on day 90 of identical experimental settings, mentioned in B. *p<0.01. Expression of CD69 on CD8^+^ T cells from lymph node of naive mice is also shown (**C**).

In addition to NLGP mediated therapeutic tumor restriction, it is also important to examine whether NLGP promotes memory response to prevent recurrence. For this experiment, eight animals have been selected with complete remission of tumor following NLGP therapy and re-inoculation with sarcoma 90 days following first tumor inoculation. Appearance of tumor was noticed in 2 among 8 animals (25%) included in this study and these two mice showed to have stable disease with average tumor size of 320 mm^3^ (Figure 6A, *diagrammatic presentation*).

Analysis of different immune compartments of NLGP-treated tumor regressed mice revealed the presence of significantly higher number of CD44^hi^CD62L^hi^ central memory CD8^+^ T cells in blood, TDLN and VDLN (Figure 6B.3, B.4). The number as well as MFI of CD44^hi^CD62L^hi^ cells was also comparatively higher in day 21 NLGP-treated sarcoma mice (Figure 6B.1, B.2). However, extent of elevation was significantly less than day 90. Besides, T cell activation marker, CD69, was also assessed and significant downregulation of CD69^+^ cells was noted in tumor free mice (Figure 6C). Both of these evidences confirmed the generation of central memory response that might prevent the tumor growth following re-inoculation on day 90.

## Discussion

Prophylactic treatment with nontoxic Neem Leaf Preparation (NLP) restricts tumor growth in mice [34]–[36]. Our sequential observations confirmed the robust immune activation by NLP [34]–[40] as well as by NLGP, an identified active principle [27]. Immune activation was correlated directly with tumor growth inhibition. However, the scenario would be different during cancer treatment. Tumor induced deviation in systemic and local immunity may confront additional challenge in effectiveness of NLGP to restrict tumor because strengthening of existing immunity is a different task from normalization of the deviated immunity. Moreover, peripherally activated T cells further exposed to TME before its anti-tumor action may thus limit functionality and durability of NLGP activated T cells.

Considering these limitations, we have selected slow growing established H-2K*^d^* sarcoma model with minimum tumor load (average TV, 62.5 mm^3^ in treatment initiation). Weekly s.c. NLGP (25 µg) injection resulted in tumor regression in 50% mice, in contrary to regression seen in 12% mice only in PBS controls. Tumor volume of mice with progressive disease in NLGP-treated group (45.8%) is significantly less from progressed mice from PBS controls (70.8%). These results suggest administration of NLGP for 4 times starting from day 7 after tumor inoculation, which inhibits tumor growth, resulting in disease stabilization or complete regression in the majority of treated animals. One surprising observation is that 25 and 50 µg dose/mice can cause tumor restriction, but 100 µg of NLGP cannot. As an immunomodulator, NLGP preferably works in lower concentration, e.g., within the range of 25–50 µg NLGP/mice, but in higher doses it fails to work. This interesting observation may open direction of our future studies, where we will attempt to know the signaling gateway of NLGP.

Involvement of CD8^+^ T cells in the therapeutic outcome of tumor host undergoing immunotherapy was discussed [41], [42] and adoptive transfer of such cells help in eradication of established tumors [43]. To evaluate the involvement of these cytotoxic cells, first, we assessed the status of CD8^+^ T cells within TILs, along with other immune organs, and upregulation was noted. These *in vivo* results are in accordance with our *in vitro* data, where NLGP showed efficacy to enhance the CD8^+^ T cells within human [21] and mouse [24] mononuclear cells. NLGP enhances antigen processing and presentation through different antigen presenting cells (APCs) and upregulates the expression of MHCs and co-stimulatory molecules [20], [22], [28]. NLGP significantly enhances the secretion of IL-12 from macrophages/dendritic cells [20] and this IL-12 regulates T cell functions, e.g. IFNγ secretion [21]. In the crosstalk between dendritic cells (DCs) and T cells, role of CD40–CD40L interaction was documented [20]. In such antigen presentation functions, involvement of helper CD4^+^ T cells is also crucial. CD4^+^ T cells play a fundamental role in the activation, expansion, and survival of primary CD8^+^ T cells [44], and they provide signals that support the establishment and maintenance of memory CD8^+^ T cells [45]. CD4 depletion in sarcoma bearing mice compromised the NLGP mediated tumor growth restriction particularly at a later stage *(data not shown)*. This data suggests the co-operation of helper cells in antitumor CD8^+^ T cell functions to restrict the tumor growth. These NLGP activated CD8^+^ T cells, then, should reach in tumor milieu to induce tumor cytotoxicity.

Consistent with this notion, we observed significant expansion of CXCR3^+^ T cells within TDLN and TIL on day 21 of NLGP-treated tumor bearer. At the same time high turnover of mRNAs of CXC ligands was detected within TME, suggesting steady pull of CXCR3^+^ T cells from TDLN to TME by ligands cxcl9-11. Dysregulated CXC receptor-ligand homeostasis in human HNSCC model was corrected by NLGP as reported [27]. Besides, upregulation of CCR5 was also detected in TDLN and TILs of NLGP-treated mice, in comparison to PBS controls. Over-expression of CCR5 on antigen presenting cells, like, monocytes/macrophages, may help to move these cells towards TME, where, they can present tumor derived peptide fragments to CD8^+^ T cells for cytotoxicity. These chemokine driven T cells were activated by NLGP, when they reached the tumor milieu through TDLN. In addition, CD8^+^ T cells remained activated as evidenced by CD69 expression in various immune organs. These NLGP activated cells also expressed greater amount of CD44 and homing receptor CD62L than PBS-treated control on day 21, which further confirmed their activation. In general after reaching tumor site, T cells become dysfunctional, because of early stage anergy, late stage exhaustion and T cell apoptosis. These T cell dysfunctions crucially associated mechanisms with immunosuppression that hinder productive antitumor immunity. Fortunately, within TME, NLGP targets T cell anergy by normalizing NFAT, DGKα, GRAIL, EGRs etc. *(unpublished observation)*. T cell exhaustion may also be targeted as we observed significant decrease in genes for tim3, pd1, lag3 and activation induced T cell apoptosis by suppressing FasR. This anti-tumor functionality within TME by NLGP might offer tumor growth restriction maximally.

We have checked functionality of CD8^+^ T cells within tumor by analyzing cells from tumors. Interestingly, all of these cells from tumor bearing NLGP-treated mice have shown greater Ki67 positivity; thus, their proliferation after tumor antigen exposure is expected. Direct involvement of CD8^+^ T cells in tumor restriction was evident by depleting these cells, as we found significant inhibition in NLGP induced tumor restriction. Our findings do not exclude the importance of other immune effectors during NLGP therapy, especially CD4^+^ T cells, although this aspect is beyond the scope of the present study.

NLGP driven CD8^+^ T cell pool participates in antitumor functions as we observed regression or minimum progression of tumors in NLGP-treated mice. IFNγ from CD8^+^ T cell creates type1 immune microenvironment. Assessment on IFNγ from different immune compartments established the greater trend of IFNγrelease from cells of draining lymph nodes and TME of NLGP-treated mice. This IFNγ potentiates several IFNγ dependent chemokines to create a pull for CD8^+^ T cell movement within tumor milieu. Accordingly, greater expression of cxcl9/10 genes, and the receptor, CXCR3, was noted in immune compartments from NLGP-treated mice. Previously, we also reported the IFNγdriven CXC chemokine signaling in NLGP-treated PBMC from HNSCC patients [28]. In addition to CXCR3, role of CCR5 signaling in T cell homing may not be ignored. Accordingly, greater expression of CCR5 and its ligands, ccl3, ccl4, ccl5 and ccl8, was demonstrated in NLGP-TME. Our data suggests that NLGP mediated upregulation of CXCR3/CCR5 is specific for T cells, not for tumor cells. Thus, NLGP may promote T cell migration, without influencing the migration of sarcoma cells. Moreover, observed CD8^+^ T cell functions are promoted by NLGP created type 1 biasness where upregulation of T-bet, along with GATA3 downregulation, was noted with overall increase in Tbet: GATA3 ratios. This type 1 microenvironment ultimately upregulates STAT1, with downregulation of STAT3, as we reported earlier in NLGP directed systems [25].

Tregs are essential for tumor-induced peripheral tolerance and are a barrier to tumor immunotherapy [46]. Some cytotoxic agents deplete Tregs systemically [47]. Inhibition of acquisition of Treg properties, downregulation of the number of CD4^+^CD25^+^Foxp3^+^ cells and withdrawal of Treg induced suppression of T cell and macrophage functions by NLGP were reported [24]. In order to discern the Treg involvement in NLGP induced promotion of T cell functions, the status of CD4^+^CD25^+^Foxp3^+^ cells was assessed. NLGP particularly promotes altered T∶Treg ratios to fasten anti-tumor effector CD8^+^ T cell functions. Inhibition of suppressor functions within tumors is associated with normalization of TME and the NLGP therapy prominently downregulates TGFβ, IL-10 and IDO within TME to promote T cell functions by suppressing Treg activity (*data not shown*). Increment in the T∶Treg ratios by maintaining the Treg pool is beneficial in preventing autoimmunity. Frequency of other immunosuppressive cellular components, MDSCs and TAM (M2 and regulatory type macrophages) is also reduced by NLGP treatment (*unpublished observation*), however, these two types of suppressor cells, here, are not included.

To further explain the *in vivo* tumor growth restriction, we have checked the cytotoxic potential of cells from NLGP-treated mice. These immune cells exhibited greater cytotoxicity than PBS controls. However, maximum killing of sarcoma cells was observed by leukocytes isolated from tumors and this finding provides explanation for tumor restriction. Expression of perforin and granzymeB is also upregulated by NLGP within TME. NLGP induces perforin-granzymeB expression in IFNγ dependent manner in T cells, but, not in NK cells [21]. On the other hand, minimum killing of lymphoma cells was detected by the same antigen experienced T cells. In correlation with the observed greater cytotoxicity by TILs, significantly higher expression of cytotoxic molecule, CD107a (*p*<0.001), was observed in TILs during their crosstalk with sarcoma cells. Zhao et al. recently reported the enhanced expression of CD107a molecules during intratumoral IL-12 gene therapy that cross prime CD8^+^ Tc1 cells reactive against tumor associated stromal antigens [48].

Although our therapeutic protocol consists of four NLGP injections following establishment of tumor, experimental studies performed here were based on studies with mice bearing day 21 tumor and sacrificed 15 days following first NLGP injection. Noted anti-tumor type 1 immunity in this point prompted us to study the memory response of CD8^+^ T cells, exhibiting remarkable tumor cell cytotoxic reactions. In initial memory experimentation, cured sarcoma bearing animals were subsequently injected with sarcoma cells 90 days after first tumor inoculation. Most of the animals (75%) rejected a sarcoma challenge, supporting therapy-induced development of a protective T cell repertoire recognizing sarcoma antigens, at the time when no palpable tumors exist. Two among 8 mice developed only small tumor on re-inoculation but their tumor volume was remarkably less than sarcoma bearing mice having no NLGP treatment. To delineate the cellular changes in the direction of memory generation, we observed significant enhancement of CD44^hi^CD62L^hi^ cells, coding central memory response, particularly in TDLN from NLGP-treated day 90 sarcoma regressed mice. A similar study on day 21 showed CD44^hi^CD62L^hi^ cellular enhancement after NLGP treatment, but that was statistically insignificant and not comparable with day 90 sarcoma regressed mice. On the contrary, the expression of CD69 was significantly less in draining lymph nodes from mice of same groups.

In summary, our data supports substantial and sustained treatment benefit(s) resulting from a weekly (7 days alternate for 4 injections total) course of NLGP injections. This therapy effectively promotes/recruits Type-1 anti-tumor CD8^+^ T cells into the TME and reduces immunologic indices associated with immune suppression, while as a consequence, approximately half of the animals receiving NLGP therapy demonstrated regressive disease. Our data supports the clinical translation of NLGP therapy, which may be also applicable for different cancer histologies. Results warrant the necessity of preclinical trial and the nontoxic NLGP therapy may be combined with low dose chemotherapy. This may be most salient in preventing or serving as a second line treatment option for patients that develop progressive disease, refractory to standard therapy. Clinicopathological studies have demonstrated a strong association between prolonged patient survival and the presence of intratumoral CD8^+^ cytotoxic T cells and an IFNγ gene signature [14], [49]. NLGP vaccination also triggers these types of T cell responses; thus, a clinical benefit might be expected.

## Materials and Methods

### Antibodies and Reagents

RPMI-1640, DMEM and Fetal Bovine Serum (FBS) were purchased from Life Technologies (NY, USA). Lymphocyte separation media (LSM) was procured from MP Biomedicals, Irvine, CA, USA. Fluorescence conjugated different anti-mouse antibodies (CD4, CD62L, CD107a, CXCR3, CCR5, GATA3, which are all FITC conjugated and CD8, CD25, CD69, CD44 and T-bet, which are all PE conjugated) were procured from either BD-Pharmingen or Biolegends (San Diego, CA, USA). Unlabeled anti-mouse antibodies were used for Foxp3 and IFNγ (BD-Pharmingen, San Diego, CA, USA). OptEIA kit (to measure IFNγ), TMB substrate solutions (for ELISA), Ki67 kit (for cell proliferation), CytoFix/CytoPerm solutions (for intracellular staining) were procured from BD-Pharmingen, San Diego, CA, USA. LDH cytotoxicity detection kit was purchased from Roche Diagnostics, Mannheim, Germany. Immunoperoxidase detection kit was obtained from Vector laboratories Inc., Burlingame, CA. RT-PCR primers were designed and procured from MWG-Biotech AG, Bangalore, India. Depleting antibody for CD8 (Clone 2.43) was purchased from Taconic (Petersburg, NY).

### NLGP

Extract of neem *(Azadirachta indica)* leaves was prepared by the method as described [34], [37]. Mature leaves of same size and color, taken from a standard source, were shed-dried and pulverized. Leaf powder was soaked overnight in PBS, pH 7.4; supernatant was collected by centrifugation at 1500 rpm 10 min. The preparation, termed neem leaf preparation (NLP), was then extensively dialyzed against PBS, pH 7.4 and concentrated by Centricon Membrane Filter (Millipore Corporation, Bedford, MA, USA) with 10 kDa molecular weight cut off. Active component of this preparation is a glycoprotein, as characterized earlier [27], termed neem leaf glycoprotein (NLGP). Protein concentration of NLGP solution was measured by Lowry's method [50]. Purity of the NLGP was confirmed by HPLC [20] before use.

### Mice and Tumors

Female Swiss mice (Age: 4–6 weeks; Body weight: 24–27 g) were obtained from the Institutional Animal Care and Maintenance Department and maintained under standard laboratory conditions. Autoclaved dry pellet diet and water were given *ad libitum*. Sarcoma 180 and lymphoma cells were maintained by regular *in vivo* intraperitoneal passage in Swiss mice and *in vitro* cultured in DMEM supplemented with 10% FBS, 2 mM L-glutamine and gentamycin (0.052 mg/ml) at 37°C. To develop solid tumors *in vivo*, Swiss mice were inoculated subcutaneously (s.c.) in right hind leg quarters with sarcoma cells (1×10^6^ cells/mice). Animal experiments were performed according to the guidelines established by the Institutional Animal Care and Ethics Committee of CNCI, Kolkata, India and this committee approved the present study.

### Tumor Growth Restriction Assay

Two groups of Swiss mice (n = 24 in each group) were inoculated with’ sarcoma (1×10^6^ cells/mice) in the lower right flank to develop solid tumors. Seven days after the tumor inoculation, mice having palpable tumors were injected with NLGP (25 µg/mice/injection) and PBS as control weekly for four times in total. Growth of solid tumor (in mm^3^) was monitored weekly by caliper measurement using the formula: (width^2^×length)/2. Mice survival was monitored regularly, till tumor size reached to 25 mm in either direction.

In addition, different doses (12, 25, 50, 100 µg), routes (subcutaneous, intravenous, intraperitoneal, intratumoral), intervals (3, 7, 15 days) and single dose on day 7 were checked.

### Isolation of Blood, Spleen, Lymph Nodes and TILs

Mice were anesthesized with ether to collect blood and, then, sacrificed by cervical dislocation to collect spleen, TDLN and VDLN aseptically. Cells were isolated from blood by centrifugal separation on LSM at 2000 rpm for 30 minutes and from organs by flashing with PBS. Isolated cells were resuspended in complete RPMI 1640. Tumors were cleaned with PBS and chopped into small pieces with scalpel. These pieces were then passed through the nylon mesh to prepare single cell suspensions. TILs were then separated from tumor cells by differential gradient centrifugation on LSM.

### CD8^+^ T Cell Purification

CD8^+^ T cells were purified from MNCs obtained from different organs and tumors, using Magnetic Associated Cell Sorter (MACS) according to instruction (Miltenyi Biotec, GmbH). In brief, isolated MNCs were labeled with Biotin-Antibody Cocktail followed by incubation with anti-Biotin microbeads. The cell suspension was then loaded on a MACS column and allowed to pass through. The effluent was collected as cell population enriched with CD8^+^ T cell fraction. The purity of cells was checked by flow cytometry after labeling with anti-CD8-PE and cell preparation with >90% purity was taken.

### Tum-Ag and TME-Ag

Peritoneally grown tumor cells were collected, stirred in 1× PBS for 2 h and centrifuged at 12,000 *g* for 1 h at 4°C. The supernatant was taken and the protein concentration was measured using Folin's phenol reagent [50] and obtained Tum-Ag stored at −20°C. On the other hand, to prepare the TME-Ag, solid tumors were chopped to prepare single cells and stirred in chilled PBS for 2 h. Antigen was prepared in similar method as used for Tum-Ag. For *in vitro* stimulation, MNCs, collected from different immune compartments, were stimulated with Tum-Ag/TME-Ag and IL-2 to generate antigen specific CTLs *in vitro*.

### Flow Cytometric Staining

Flow cytometric analysis for T cell surface phenotypic markers was performed after labeling of 1×10^6^ cells with different anti-mouse fluorescence labeled antibodies (CD4, CD8, CD25, CD44, CD62L, CD69, CXCR3 and CCR5) as per manufacturer's recommendation. Sarcoma cells were also stained for CXCR3 and CCR5 expression. After labeling, cells were washed in FACS buffer (PBS with 1% FBS). Similarly, intracellular molecules, IFNγ, Foxp3, T-bet and GATA3, were stained with anti-mouse fluorescence labeled antibodies using cytofix–cytoperm reagents as per manufacturer's recommendation. For CD107a mobilization, an assay was performed along with surface staining for CD8 by the method reported [51]. Isolated cells from TDLN and tumors were stimulated with Tum-Ag for 48 h. Sarcoma cells were plated on a 96 well U bottom culture plate for 4 h and incubated with stimulated T cells (E∶T = 5∶1) for 5 h at 37^°^C, 5% CO_2_ with monensin (GolgiStop; BD Biosciences). Cells were stained with anti-CD107a mAbs.

In all flow cytometric staining, cells were fixed with 1% paraformaldehyde in PBS and cytometry was performed with Cell Quest software on a FACSCaliber (Becton Dickinson, Mountainview, CA). Suitable negative isotype controls were used to rule out the background. Percentages of each positive population and MFI were determined by using quadrant statistics.

### Ki67 Staining

Ki67 is expressed in proliferating cells [52]. The proliferation of T cells obtained from tumors was assessed for expression of Ki67. The cells were pelleted down and 70–80% chilled ethanol was added to the pellet (1–5×10^7^ cells) with vortexing and then incubated at −20°C for 2 h. The cells were washed twice with staining buffer and centrifuged for 10 min at 200 *g*. Cells were then resuspended to a concentration of 1×10^7^ cells/ml. Cell suspension was then transferred into each sample tube to add anti-Ki67 antibody and mixed gently. The tubes were incubated at room temperature for 20–30 min in dark. Cells were washed and 0.5 ml of staining buffer was added to each tube and analyzed flow cytometrically.

### ELISA

To quantify IFNγ, supernatants from MNC cultures containing T cells were harvested after 48 h and filtered (0.2 µm filters). IFNγ secretion was assessed with ELISA kits as per manufacturer's instruction and optical density was measured at 450 nm using microplate reader (BioTek Instruments Inc., Vermont, USA).

### RT-PCR

mRNA was isolated from TILs and TDLN cells and cDNA was synthesized by reverse transcription. Amplification was done by primer specific polymerization. Finally, the expression was detected by running the PCR products in agarose gel. The primer sequences of mouse genes, cxcr3, ccl9, ccl10, ccr5, ccl3, ccl4, ccl5, ccl8, granzyme B, perforin and β-actin, are presented in Table 2.

**Table 2:** Primer sequences of various genes studied.

Name	Primer sequence (5′-3′)	Product size
CXCR3-forward	GCTGCTGTCCAGTGGGTTTT	67 bp.
CXCR3-reverse	AGTTGATGTTGAACAAGGCGC	
CXCL9-forward	TGGGCATCATCTTCCTGGAG	204 bp.
CXCL9-reverse	CCGGATCTAGGCAGGTTTGA	
CXCL10-forward	CCAAGTGCTGCCGTCATTTT	177 bp.
CXCL10-reverse	CTCAACACGTGGGCAGGATA	
CCR5-forward	ACTGCTGCCTAAACCCTGTCA	78 bp.
CCR5-reverse	GTTTTCGGAAGAACACTGAGAGATAA	
CCL3-forward	CCTCTGTCACCTGCTCAACA	163 bp.
CCL3-reverse	GATGAATTGGCGTGGAATCT	
CCL4-forward	GCTGTGGTATTCCTGACCAAA	196 bp.
CCL4-reverse	AAATCTGAACGTGAGGAGCAA	
CCL5-forward	CCCTCACCATCATCCTCACT	186 bp.
CCL5-reverse	TCCTTCGAGTGACAAACACG	
CCL8-forward	ACGCTAGCCTTCACTCCAA	231 bp.
CCL8-reverse	TCTGGAAAACCACAGCTTCC	
Perforin-forward	GATGTGAACCCTAGGCCAGA	161 bp.
Perforin-reverse	GGTTTTTGTACCAGGCGAAA	
Granzyme B-forward	TCGACCCTACATGGCCTTAC	198 bp.
Granzyme B-reverse	TGGGGAATGCATTTTACCAT	
TIM3-forward	CCCCTGCCAAGTACTCATGT	161 bp.
TIM3-reverse	CAAGTGCCCCAGGTGTAGAT	
PD1-forward	CATGCCCAGGTACCTCAGTT	236 bp.
PD1-reverse	GAACCCAACTCCAGGACAGA	
LAG3-forward	TTCCGGCTCAGAGGAAGATA	200 bp.
LAG3-reverse	TTTTTGATGCTGCTGACAGG	
β-Actin-forward	CAACCGTGAAAAGATGACCC	228 bp.
β-Actin-reverse	ATGAGGTAGTCTGTCAGGTC	

PCR products were identified by image analysis software for gel documentation (BioRad, Hercules, CA) followed by electrophoresis on 1.5% agarose gels and staining with ethidium bromide.

### Immunohistochemistry

Sections (3 µm) from solid tumors were mounted on poly-L-lysine coated slides and allowed to dry at 37°C, followed by incubation in an incubator at 60°C for 1 h. After deparaffinization and rehydration, tissue sections were retrieved for antigens using 0.05 (M) borate buffer, pH 8 at 80°C for 45 min. Afterwards, endogenous peroxidase was inhibited with 0.3% H_2_O_2_ and blocked with 5% BSA in a humid chamber for 30 min each, followed by incubation with primary mouse anti-CD8 antibody (Biolegend, clone 53–6.7). After three PBS rinses slides were incubated with the HRP conjugated secondary antibody for 30 min. Tissue staining was visualized with an AEC substrate solution (Vector laboratories Inc., Burlingame, CA) and counterstained with Mayer's hematoxylin and mounted. Using a mouse IgG performed negative controls.

### Cytotoxicity Assay

Cellular cytotoxicity was determined by measuring lactate dehydrogenase (LDH) released by target cells using a commercially available kit (Roche Diagnostics, Mannheim, Germany).

### Statistical Analysis

All results represent the average of separate *in vivo* and *in vitro* experiments. Number of experiments is mentioned in the Result section and legends to figures. In each experiment a value represents the mean of three individual observations and is presented as mean±standard deviation (SD) or standard error (SE) and SD<0.05 is considered significant. Statistical significance was established by Tukey: Comparison of all pairs of columns test based on one-way ANOVA using INSTAT 3 Software.
